# Salmonella polarises peptide-MHC-II presentation towards an unconventional Type B CD4^+^ T-cell response

**DOI:** 10.1002/eji.201242983

**Published:** 2013-01-14

**Authors:** Nicola P Jackson, Yu Hui Kang, Nicolas Lapaque, Hans Janssen, John Trowsdale, Adrian P Kelly

**Affiliations:** 1 Department of Pathology, University of CambridgeCambridge, United Kingdom; 2 Division of Cell Biology, The Netherlands Cancer InstituteAmsterdam, The Netherlands

**Keywords:** Autoimmunity, Bacterial Infections, CD4 T cells, Tolerance

## Abstract

Distinct peptide-MHC-II complexes, recognised by Type A and B CD4^+^ T-cell subsets, are generated when antigen is loaded in different intracellular compartments. Conventional Type A T cells recognize their peptide epitope regardless of the route of processing, whereas unconventional Type B T cells only recognise exogenously supplied peptide. Type B T cells are implicated in autoimmune conditions and may break tolerance by escaping negative selection. Here we show that *Salmonella* differentially influences presentation of antigen to Type A and B T cells. Infection of bone marrow-derived dendritic cells (BMDCs) with *Salmonella enterica serovar Typhimurium* (*S. Typhimurium*) reduced presentation of antigen to Type A T cells but enhanced presentation of exogenous peptide to Type B T cells. Exposure to *S. Typhimurium* was sufficient to enhance Type B T-cell activation. *Salmonella Typhimurium* infection reduced surface expression of MHC-II, by an invariant chain-independent trafficking mechanism, resulting in accumulation of MHC-II in multi-vesicular bodies. Reduced MHC-II surface expression in *S. Typhimurium*-infected BMDCs correlated with reduced antigen presentation to Type A T cells. S*almonella* infection is implicated in reactive arthritis. Therefore, polarisation of antigen presentation towards a Type B response by S*almonella* may be a predisposing factor in autoimmune conditions such as reactive arthritis.

## Introduction

*Salmonella enterica* is an intracellular pathogen that survives and replicates in phagocytic cells within specialised compartments known as *Salmonella*-containing vacuoles (SCV) [1]. Following oral ingestion, *Salmonella* crosses the intestinal epithelium by invasion of non-phagocytic enterocytes or via M cells overlying Peyer's Patches [2]. Alternatively, *Salmonella* is directly taken up by DCs that intercalate between intestinal epithelial cells [3]. *Salmonella* can disseminate extracellularly or be engulfed by macrophages in the submucosa [2]. *Salmonella* pathogenicity islands (SPI) are critically important for virulence. They encode type III secretion systems (T3SS) that inject bacterial effector proteins into host cells. T3SS-1 is encoded within SPI1 and is required for invasion of host cells, whereas T3SS-2 is encoded by SPI2 and contributes to immune evasion and maintenance of the SCV by intracellular *Salmonella* [4]. *Salmonella enterica* serovars such as *Typhimurium* (*S. Typhimurium*) and Enteritidis cause rapid-onset gastroenteritis in a range of species, whereas serovars such as Typhi and Paratyphi cause systemic typhoid fever in humans. *Salmonella* Typhi can establish life-long infection of the gall bladder in 1–4% of patients. These typhoid carriers exhibit normal antibody responses to *Salmonella* Typhi antigens but have an impaired cell-mediated immune response [5].

MHC-II molecules play an essential role in the cell-mediated immune response by presenting antigenic peptides to CD4^+^ T cells. Immature MHC-II molecules are assembled in the ER and are composed of α and β chains in complex with preformed trimers of invariant chain (Ii) [6]. Ii occupies the peptide-binding groove of MHC-II to prevent premature peptide binding and chaperones the MHC-II complex from the ER to the endocytic pathway. Entry into the endocytic pathway is predominantly by clathrin-mediated endocytosis from the plasma membrane [7], but can also be direct from the trans-golgi network [8]. Once inside the endosomal compartments, Ii is degraded by lysosomal proteases until only CLIP is left bound in the MHC-II peptide-binding groove. HLA-DM exchanges CLIP for antigenic peptides in late endosomal compartments and mature peptide-MHC-II (pMHC-II) complexes are then exported to the cell surface [9]. In DCs, ubiquitination of a conserved lysine residue in the β chain cytoplasmic tail regulates surface expression and targeting of pMHC-II into late endosomal multi-vesicular bodies (MVBs) [10].

Formation of pMHC-II conformers from native protein occurs primarily in HLA-DM^+^ late endosomes and generates stable complexes that are recognised by conventional Type A CD4^+^ T cells. In contrast, loading of exogenous peptide can occur throughout the endosomal pathway or at the cell surface and can generate pMHC-II conformers that are recognised by conventional Type A and unconventional Type B CD4^+^ T cells [11]. Type B T cells only recognise exogenous peptide and not the identical peptide when processed from protein. As a consequence, Type B T cells escape negative selection and are implicated in autoimmune conditions. In the NOD mouse model, Type B insulin-reactive T cells are pathogenic and trigger diabetes in adoptive transfer experiments [12]. Type B T cells constitute 30–50% of the T-cell repertoire [13], and phenotypically may resemble either Th1 or Th2 CD4^+^ T cells [12].

*Salmonella* is reported to interfere with MHC-II antigen processing and presentation to CD4^+^ T cells [14–17]. The relevance of these mechanisms in vivo is not clear as CD4^+^ T-cell priming has also been observed in mouse models of *Salmonella* infection [18–21]. We have previously shown that *Salmonella* infection of human DCs results in polyubiquitination and reduced surface expression of MHC-II [15, 22]. In this study, we investigate how *Salmonella* influences MHC-II trafficking and presentation of antigen to Type A and B CD4^+^ T cells.

## Results

### MHC-II accumulates in MVBs in *Salmonella*-infected cells

MHC-II is specifically removed from the surface of *Salmonella*-infected cells and accumulates in intracellular vesicles that resemble HLA-DM^+^ LAMP-1^+^ EEA^−^ peptide-loading compartments [15, 22]. To better define the nature of these compartments, MHC-II localisation was assessed in *Salmonella*-infected MelJuSo cells, as the endocytic pathway is well characterised in this human epithelial-like melanoma cell line [23]. Cell surface HLA-DR was labelled with the monoclonal antibody L243 and after internalisation was visualised by cryo-immunoelectron microscopy.

**Figure 1:**
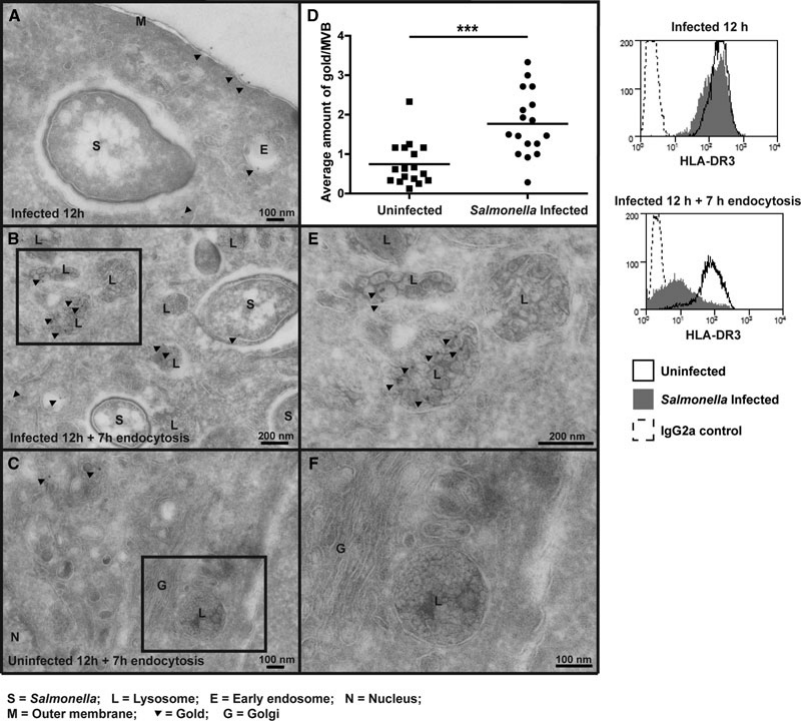
MHC-II accumulates in MVBs in *Salmonella*-infected cells. MelJuSo were infected for 20 min with invasive GFP-*S. Typhimurium* (MOI 50). Cell surface MHC-II was labelled (L243) at 12 h post-infection and then cells were fixed (A) or further incubated until 20 h post-infection before fixation (B, C, E and F). Cell sections were processed for cryo-immunoelectron microscopy and HLA-DR localisation was visualised with Protein A-gold (10 nm). (D) Graph represents average amount of gold (HLA-DR)/MVB in each cell analysed. Average amount of gold/MVB was calculated for at least 15 cells per condition and comparison of distributions was assessed by unpaired two-tailed *t*-test. Boxed areas from (B) and (C) are magnified twofold in (E) and (F), respectively. Histograms show surface HLA-DR measured by flow cytometry in infected and uninfected MelJuSo at time points indicated. Refer to Supporting Information Fig. 1A for gating strategy. Data are representative of two independent experiments.

HLA-DR was predominantly detected at the cell surface at 12 h post-infection in both uninfected (data not shown) and *Salmonella*-infected cells (Fig. 1A). Between 12 and 20 h post-infection, HLA-DR was endocytosed and distributed within early endosomes, MVBs and at the cell surface in uninfected cells (Fig. 1C and F). In *Salmonella*-infected cells, there was a twofold greater accumulation of HLA-DR in MVBs compared with uninfected cells (Fig. 1B, D and E). The internalised MHC-II was not significantly associated with the SCV but localised to MVBs that most likely represent conventional MHC-II containing compartments found in the *Salmonella*-infected cells. There were fewer MVBs in uninfected cells suggesting that *Salmonella* may enlarge this compartment through accumulation of intracellular HLA-DR (data not shown). Since *Salmonella* infection results in polyubiquitination of MHC-II, and ubiquitination regulates sorting of MHC-II at MVBs [10, 15], these results may suggest that *Salmonella*-induced ubiquitination of MHC-II enhances accumulation in MVBs to prevent recycling of mature MHC-II to the cell surface.

### MHC-II down-regulation by *Salmonella* requires clathrin but not invariant chain-directed trafficking

**Figure 2:**
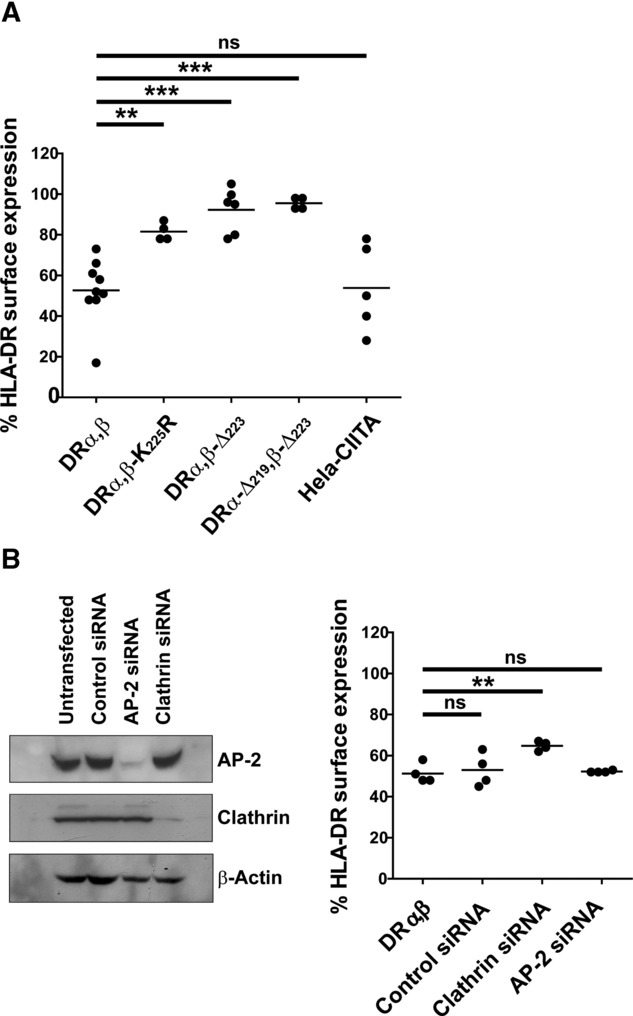
MHC-II down-regulation by *Salmonella* requires clathrin but not invariant chain-directed trafficking. (A) HeLa cells stably expressing HLA-DR WT (DRα,β) and cytoplasmic tail mutants were generated. HLA-DR surface expression was assessed by flow cytometry at 20 h post-infection with invasive GFP-*S. Typhimurium* and compared with HeLa-CIITA (Ii positive) cells. Refer to Supporting Information Fig. 1A and B for gating strategy and representative flow cytometry data. Graph shows percent of normal HLA-DR surface expression in uninfected (GFP-negative) cells combined from at least four independent experiments. (B) HeLa cells stably expressing HLA-DR WT (DRα,β)(Ii negative) were transfected with AP-2, clathrin or control siRNAs. Cells were infected with invasive GFP-*S. Typhimurium* after 5 days of AP-2 or clathrin depletion and surface HLA-DR was assessed as described in (A). Western blot shows AP-2 and clathrin depletion from representative cell lysates after 5 days of siRNA treatment. The loading control is β-actin. Graph shows percent of normal surface HLA-DR expression in uninfected (GFP negative) cells combined from four independent experiments. Comparison of distributions was performed by unpaired (A) or paired (B) two-tailed *t*-tests.

To determine whether Ii-directed trafficking of MHC-II is required by *Salmonella* to regulate MHC-II surface expression, we generated HeLa cell transfectants stably expressing HLA-DR, but lacking endogenous Ii. There was no significant difference in the extent of HLA-DR down-regulation by *Salmonella* in HeLa cells expressing CIITA (Ii-positive) and HeLa cells transduced with HLA-DR (Ii-negative) (Fig. 2A). As expected, HLA-DR dimers that lacked the DRβ cytoplasmic tail (DRα-Δ_219_,β-Δ_223_ and DRα,β-Δ_223_) or with a lysine to arginine mutation in the β chain ubiquitination site (DRα,β-K_225_R), were not down-regulated by *Salmonella* (Fig. 2A).

Endocytosis of pMHC-II is clathrin, AP-2 and dynamin independent [24]. To examine whether HLA-DR down-regulation by *Salmonella* requires AP-2 and clathrin, Ii-negative HeLa cells stably expressing HLA-DR were transfected with AP-2 and clathrin siRNA oligonucleotides and surface expression of HLA-DR was assessed by flow cytometry. In the absence of Ii, siRNA knockdown of clathrin, but not AP-2, reduced HLA-DR down-regulation by *Salmonella* (Fig. 2B, right panel). These data show that down-regulation of pMHC-II surface expression by *Salmonella* requires clathrin but was independent of Ii-directed trafficking and AP-2.

### Salmonella down-regulates murine MHC-II surface expression and antigen presentation to CD4^+^ T cells

**Figure 3:**
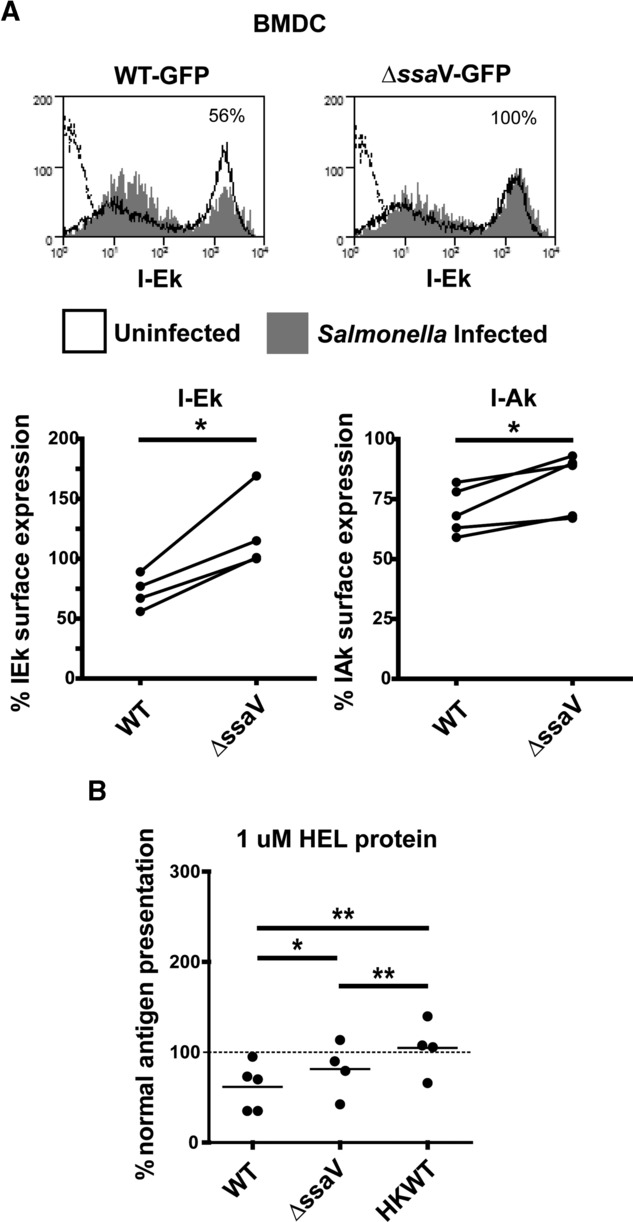
*Salmonella* downregulates I-A and I-E surface expression and presentation of antigen to CD4^+^ T cells. (A) BMDCs were infected with opsonised GFP-*S. Typhimurium* (MOI 10) then I-A^k^ (OX6) and I-E^k^ (14.4.4s) surface expression was compared in infected (GFP positive) and uninfected (GFP negative) CD11c/CD11b^+^ BMDCs by flow cytometry. Refer to Supporting Information Fig. 1A for gating strategy. Histograms (upper panels) show I-E^k^ surface expression in infected and uninfected BMDCs from a representative of at least four independent experiments. Graphs (lower panels) show percent of normal (GFP negative) I-A^k^ or I-E^k^ surface expression combined from four independent preparations of BMDCs infected with WT or SPI2-deficient (Δ*ssa*V) *S. Typhimurium*. (B) BMDCs (in triplicate) were uninfected or infected with opsonised WT, HKWT or Δ*ssa*V *S. Typhimurium* (MOI 10). From 20 h post-infection, cells were incubated with HEL protein and Type A CD4^+^ T hybridoma cells (3A9) at a ratio of 5 T cells: 1 BMDC. After 24 h, culture supernatants were harvested and T-cell activation was quantified by IL-2 ELISA. Graph shows percent of normal mean (uninfected) I-A^k^-dependent HEL presentation to Type A T cells combined from at least four independent experiments. Antigen presentation in uninfected BMDCs is shown as a dashed line. Comparison of distributions was performed by paired two-tailed *t*-tests.

To examine the effect of *Salmonella* on T-cell presentation, we first exposed murine BMDCs to GFP-expressing *Salmonella* and examined surface I-A and I-E expression. Exposure to *Salmonella* increased overall I-A^k^ and I-E^k^ surface expression, consistent with BMDC activation and maturation [25] (data not shown). Infection of BMDCs with WT *Salmonella* reduced surface expression of I-A^k^ and I-E^k^. I-A^k^ and I-E^k^ down-regulation was not detected following infection with Δ*ssa*V *Salmonella* (Fig. 3A), as observed previously for HLA-DR [15]. This indicated that the SPI2 effector system was also required to regulate MHC-II surface expression in murine cells. Comparable I-A and I-E down-regulation was also seen for the b and d haplotypes (data not shown). MHC-II down-regulation was not detected in either human monocyte-derived macrophages, or a murine macrophage cell line, RAW264.7-CIITA (Supporting Information Fig. 2). The reason for this is unknown but may reflect functional differences between DCs and macrophages [26].

To assess antigen presentation in the context of *Salmonella* infection we analysed I-A^k^-dependent presentation of the model antigen hen egg lysozyme (HEL) by BMDCs to a CD4^+^ T-cell hybridoma expressing a HEL-specific TCR (3A9). T-cell hybridomas do not require co-stimulation and therefore pMHC-II levels should directly correlate with the extent of antigen presentation. BMDCs were used because they can be generated in large quantities and they resemble the myeloid CD11b^+^ DCs present in the sub-epithelial dome of murine Peyer's patches where *Salmonella* internalise early after oral infection in vivo [3, 27].

Incubation of BMDCs with exogenous HEL protein resulted in dose-dependent HEL-specific T-cell activation, as measured by IL-2 production (data not shown). After infection of BMDCs with *Salmonella* a reduction in T-cell activation was observed (Fig. 3B), in line with previous observations using exogenous antigen [14, 28]. The reduction in T-cell activation was SPI2 dependent, although the effect was subtle. A MOI of ten bacteria to one BMDC was used as this does not induce significant NO production by the BMDCs (confirmed by Griess assay; data not shown) [14]. Infection with an equal number of heat-killed (HK) *Salmonella* had no influence on T-cell activation confirming that viable bacteria are required to inhibit antigen presentation in the absence of NO. These data show that down-regulation of MHC-II surface expression by *Salmonella* correlates with reduced presentation of antigen to CD4^+^ T cells.

### Salmonella enhances presentation of exogenous peptide to Type B CD4^+^ T cells

To determine whether *Salmonella* also influenced presentation of antigen to Type B CD4^+^ T cells, we compared I-A^k^-dependent presentation of exogenous HEL protein and HEL_46–61_ peptide by BMDCs to a Type A T-cell hybridoma (3A9) and a Type B T-cell hybridoma (11A10) with identical peptide specificity.

**Figure 4:**
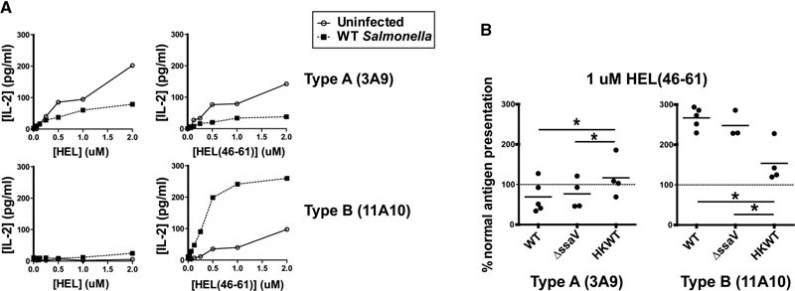
*Salmonella* infection enhances presentation of exogenous peptide to Type B T cells. BMDCs (in triplicate) were infected with opsonised WT (A and B), SPI2-deficient (Δ*ssa*V) (B) or HKWT (B) GFP-*S. Typhimurium* (MOI 10). From 20 h post-infection, cells were incubated with HEL protein or HEL_46–61_ peptide and 3A9 (Type A) or 11A10 (Type B) T hybridoma cells at a ratio of 5 T cells: 1 BMDC. After 24 h, culture supernatants were harvested and T-cell activation was quantified by IL-2 ELISA. (A) Graphs show mean IL-2 concentration from a representative of at least four independent experiments. Error bars represent SD. (B) Graphs show percent of normal (uninfected) I-A^k^-dependent HEL_46–61_ presentation to Type A or B T cells combined from at least three independent experiments. Antigen presentation in uninfected BMDCs is shown as a dashed line. Comparison of distributions was performed by paired two-tailed *t*-tests.

In line with previous publications, incubation of BMDCs with exogenous HEL protein or HEL_46–61_ peptide resulted in dose-dependent HEL-specific Type A T-cell activation, whereas only incubation with exogenous HEL_46–61_ peptide resulted in equivalent activation of Type B T cells (Fig. 4A, open circles) [11]. Infection of BMDCs with WT *Salmonella* inhibited presentation of both exogenous HEL protein and HEL_46–61_ peptide to Type A T cells. Intriguingly, WT *Salmonella* infection caused a dramatic increase in the presentation of exogenous HEL_46–61_ peptide to Type B T cells, but had little effect on presentation of HEL protein (Fig. 4A). Unlike inhibition of Type A T-cell activation by *Salmonella*, enhanced presentation of HEL_46–61_ peptide to Type B T cells was not SPI2 (*ssa*V) dependent (Fig. 4B). Furthermore, infection with an equal number of HK *Salmonella* had no effect on Type A T-cell activation but subtly increased Type B T-cell activation (Fig. 4B). These data show that *Salmonella* influenced antigen presentation in several distinct ways. Most dramatically *Salmonella* infection resulted in elevated presentation of exogenous peptide to Type B T cells. This was associated with a reduction in presentation of peptide or protein antigen to Type A T cells.

### Exposure to *Salmonella* is sufficient to enhance presentation of exogenous peptide to Type B T cells

At the MOI used in the above experiments (MOI = 10), only 10–20% of the BMDCs were infected with *Salmonella*. This suggested that direct infection may not be required and that a soluble factor produced by infected BMDCs could be influencing neighbouring cells. To screen for potential soluble factors produced by *Salmonella*-infected BMDCs, culture supernatant was harvested from infected BMDCs at 20 h post-infection and incubated with fresh BMDCs, Type B T hybridoma cells and HEL_46–61_ peptide.

**Figure 5:**
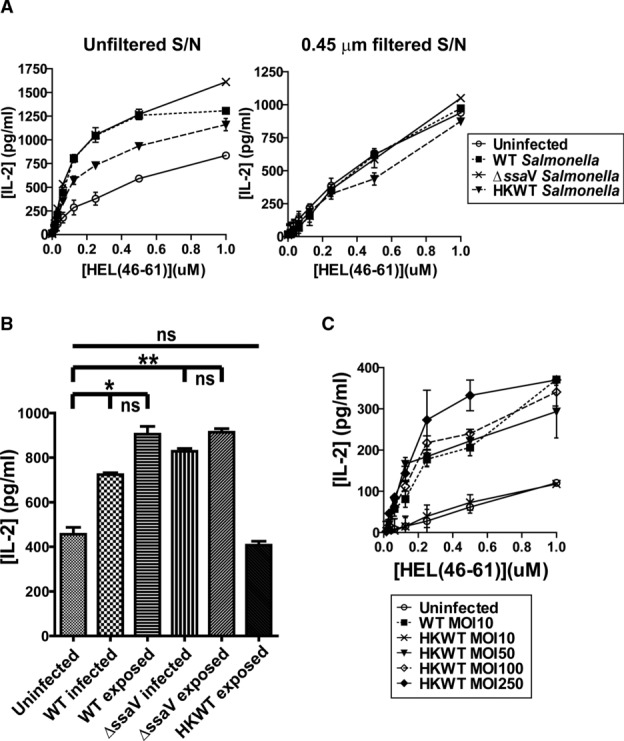
Exposure to *Salmonella* is sufficient to enhance presentation of exogenous peptide to Type B T cells. BMDCs (in triplicate) were infected with opsonised WT, SPI2-deficient (Δ*ssa*V) or HKWT GFP-*S. Typhimurium* (MOI 10, unless specified (C)). For antigen presentation, BMDCs were incubated with HEL_46–61_ peptide and 11A10 (Type B) T hybridoma cells at a ratio of 5 T cells: 1 BMDC. After 24 h, culture supernatants were harvested and T-cell activation was quantified by IL-2 ELISA. (A) At 20 h post-infection, culture supernatant was harvested and incubated with fresh BMDCs, HEL_46–61_ peptide and 11A10 (Type B) T cells. Where indicated, culture supernatant was filtered (0.45 μm) prior to incubation with fresh BMDCs. (B) At 20 h post-infection, GFP-*S. Typhimurium*-infected BMDCs were sorted from the exposed but uninfected population. Refer to Supporting Information Fig. 1A for representative gating strategy. Presentation of 0.5 μM HEL_46–61_ peptide to 11A10 (Type B) T cells was compared for BMDCs that were unexposed, exposed but uninfected, or infected with *S. Typhimurium*. Comparison of distributions was performed by unpaired two-tailed *t*-tests. (A–C) Data shown are the mean + SD and are representative of one out of at least four independent experiments.

Incubation of fresh BMDCs with culture supernatant from *Salmonella-*infected BMDCs was sufficient to enhance presentation of exogenous peptide to Type B T cells (Fig. 5A). Clearance of the supernatant using a 0.45 μm filter prior to incubation with fresh BMDCs (Fig. 5A) or separation of infected BMDCs from fresh BMDCs and T cells using 0.45 μm transwells (Supporting Information Fig. 4) abrogated the effect. This suggested that whilst a component of culture supernatant from *Salmonella-*infected BMDCs can influence uninfected BMDCs in *trans*, the factor responsible was not smaller than 0.45 μm.

*Salmonella* are rod-shaped bacteria, 0.5–1.5 μm in diameter and 2–5 μm in length. Clearance of culture supernatant using a 0.45 μm filter would therefore remove any intact *Salmonella* present. To determine whether direct exposure of BMDCs to *Salmonella* was sufficient to enhance presentation of exogenous peptide to Type B T cells, BMDCs were infected with GFP-expressing *Salmonella* and then sorted at 20 h post-infection to separate infected from exposed but uninfected populations. Presentation of HEL_46–61_ peptide to Type B T hybridoma cells was compared for BMDCs that were unexposed, exposed but uninfected, or exposed and infected with *Salmonella*, respectively.

Exposure of BMDCs to *Salmonella* was sufficient to enhance presentation of exogenous peptide to Type B T cells (Fig. 5B). There was no significant difference in the extent of Type B T-cell activation between sorted BMDCs that were exposed to, or infected with *Salmonella*. There was a consistent trend towards reduced presentation in the infected BMDCs, although the effect was subtle (Fig. 5B). Notably, presentation of peptide to Type B T cells was enhanced by HK *Salmonella* if the MOI was increased (Fig. 5C). This suggested that whilst viable bacteria contribute more significantly to the enhanced presentation of exogenous HEL_46–61_ peptide to Type B T cells observed, viability is not essential.

## Discussion

We show that *Salmonella* infection influences MHC-II antigen presentation to CD4^+^ T cells by two distinct mechanisms. Intra-cellular replication of *Salmonella* resulted in reduced expression of pMHC-II complexes at the cell surface and altered presentation of antigen to CD4^+^ T cells. Most importantly, exposure of BMDCs to *Salmonella* resulted in enhanced presentation of exogenous peptide to Type B CD4^+^ T cells, which have been linked to auto-immune disease progression [12].

We first examined the influence of intracellular *Salmonella* on MHC-II trafficking and localisation. Using Ii-negative HeLa cells, we showed that down-regulation of MHC-II by *Salmonella* was independent of Ii-directed trafficking and AP-2, but required clathrin. As AP-2 is the principal adaptor protein required for formation of clathrin-coated pits at the plasma membrane [29], it is unlikely that MHC-II down-regulation by *Salmonella* requires the formation of clathrin-coated pits. Distinct clathrin coats are also present at the cytoplasmic face of MVBs and are proposed to concentrate cargo for subsequent incorporation into luminal vesicles [29]. In addition, sorting of pMHC-II into luminal vesicles at MVBs is regulated by ubiquitination [30]. Therefore, the requirement for clathrin by *Salmonella* may be related to clathrin-dependent sorting of ubiquitinated cargo at MVBs.

We next confirmed that *Salmonella* down-regulated surface expression of I-A and I-E in BMDCs, similar to what has been observed in human cells [15, 22]. This validated the use of murine T-cell reagents to assess antigen presentation following *Salmonella* infection of BMDCs. *Salmonella* infection of murine DCs has been reported to inhibit presentation of antigen to CD4^+^ T cells [14, 28]. Here we compared the influence of *Salmonella* on presentation of antigen to Type A and B CD4^+^ T-cell subsets. In contrast to the suppressive effect of *Salmonella* infection on presentation of both exogenous protein and peptide antigen to Type A T cells, presentation of peptide to Type B T cells was significantly enhanced. *Salmonella* infection did not significantly alter presentation of exogenous protein antigen to Type B T cells. This contrasts with the recent data of Strong and Unanue showing that TLR ligands have no effect on the presentation of peptide via the Type B conformer in splenic DCs [31]. This may reflect differences in antigen handling between DC subsets as reported by Lovitch et al. for presentation of native antigen to Type B T cells using LPS-stimulated BMDCs [32].

The relevance of reduced Type A presentation in relation to immunity to *Salmonella* infection in vivo is not clear. Whilst chronic typhoid carriers exhibit impaired humoral and cellular immunity [5], rapid priming of CD4^+^ T cells in mouse models of *Salmonella* infection was reported to elicit effective Th1 responses [21]. Regardless, priming of Type B T-cell responses does not imply any perturbation in the Th1/Th2 balance and could occur in the presence or absence of effective anti-*Salmonella* responses. Taken together, these data suggest that *Salmonella* infection polarises antigen presentation by stabilising or enhancing formation of the pMHC-II conformer recognised by Type B T cells, leading to increased Type B T-cell activation. In heavily infected BMDCs, this polarisation may be further promoted by reduced presentation of pMHC-II conformers recognised by Type A T cells.

Mere exposure to HK *Salmonella* or supernatant from *Salmonella*-infected BMDCs was sufficient to enhance presentation of exogenous peptide to Type B T cells. This phenotype is unlikely to be caused by soluble TLR ligands such as LPS and flagellin, as these are shed by *Salmonella* in significant amounts [33] and the effect was lost when the culture supernatant was filtered or when infected BMDCs were spatially separated from uninfected BMDCs and T cells. It is unlikely to be due to secretion of a soluble cytokine as this would also remain in the filtered supernatants. Direct contact between *Salmonella* and BMDCs is required. We are currently attempting to identify the bacterial components responsible for this effect.

The potential for Type B T-cell activation to lead to auto-immune disease is established [12]. Infection of the gastrointestinal tract with *Salmonella*, as well as *Yersinia, Campylobacter* and *Shigella* [34], is frequently associated with reactive arthritis in humans [35] and mice [36]. *Salmonella* infection in humans caused incidence rates of between 6 and 30% (with variable severity), reflecting the propensity of different *Salmonella* species to induce arthritis. The enhanced activation of Type B T cells observed with *Salmonella* infection is not limited to pathogen-specific T cells as the antigens used in these experiments were not derived from *Salmonella*. Therefore, it is possible that infected DCs in vivo may incidentally present self-peptide-associated Type B conformers, leading to activation of potentially autoreactive Type B T cells. Processes such as inflammation may increase the supply of exogenous peptide and thereby facilitate the generation of Type B pMHC-II conformers. In fact, several immune cell types, including neutrophils [37] and DCs [38, 39], are known to generate exogenous antigenic peptides. In Type 1 diabetes, related mechanisms are thought to generate peptides from the insulin B chain, which when presented on MHC-II are specifically recognised by diabetogenic Type B T cells, leading to disease [12].

This study is the first to show that exposure of BMDCs to *Salmonella* enhances the presentation of exogenously supplied peptides to Type B T cells. It suggests a mechanism by which *Salmonella* infection could lead to a breakdown in immunological tolerance. Further studies will be required to identify the factor responsible for this alteration in peptide presentation and to evaluate the role of Type B T cells in infection and autoimmunity.

## Materials and methods

### Antibodies

Antibodies were from Thermo Scientific: rabbit anti-mouse IgG-Fc RPE; Dako: rabbit anti-mouse Igs/HRP; BD Transduction Laboratories: mouse anti-human AP-50 (611350), mouse anti-human clathrin heavy chain (610499); Sigma: mouse anti-human β-actin (AC-74); eBioscience: anti-mouse CD11c PE-Cy5 (N418), anti-mouse I-E^d/k^ PE (14.4.4s); GeneTex: anti-mouse I-A^k^ R-PE (OX-6). The anti-HLA-DR antibody was from clone L243 and is specific for peptide-loaded HLA-DR.

### Plasmid constructs

pCMV8.91, pMD-G and pHRSin-cPPT-SGW lentiviral constructs were provided by Paul Lehner (Cambridge, UK). The HLA-DR3 sequences [15] were cloned into the *Bam*HI and *Not*I sites of pHRSin-cPPT-SGW after eGFP was excised.

### Cell culture, lentiviral transduction and siRNA transfection

HEK293-T, MelJuSo, RAW264.7-CIITA, HeLa and HeLa-CIITA cells were maintained in DMEM, 10% FCS. 3A9 and 11A10 T-cell hybridomas were maintained in DMEM, 5% FCS. Monocyte-derived macrophages and serum were prepared from PBMCs isolated from Buffy Coats (British National Transfusion Service) using Lymphoprep (Axis-Shield). Serum was filtered and heat-inactivated. Monocytes were differentiated into macrophages for 7 days in RPMI-1640 (Sigma), 3% autologous serum, 50 ng/mL M-CSF (Peprotech). For lentivirus production, HEK293-T cells were transfected with pCMV8.91, pMD-G and pHRSin-cPPT-SGW using polyethylenimine (Sigma Aldrich, UK) [40]. After 48 h, lentivirus-containing supernatants were filtered (0.2 μm) and applied to HeLa cells. Transduced cells were sorted using a MoFlo flow cytometer (Cytomation). For siRNA transfection, HeLa were seeded in 6-well plates and transfected with siRNA oligonucleotides using Oligofectamine (Invitrogen). Cells were reseeded at 48 h post-transfection before a second transfection with the same oligonucleotide. siRNA oligonucleotides for AP-2 and clathrin heavy chain were from Qiagen [15].

### BMDC preparation and antigen presentation

Mice were maintained according to institutional guidelines at the University of Cambridge. BM was harvested from femurs/tibias of 8–12 week female C3H/HeNCrl mice (Charles River) and passed through a 70 μm strainer in IMDM. BM cells were seeded in 9 cm plates at 1 × 10^6^ cells/mL in IMDM, 10% FCS, 2 mM Ultraglutamine (Lonza), 10 ng/mL IL-4 (Peprotech), 20 ng/mL GM-CSF (Peprotech) and penicillin/streptomycin (PAA Laboratories) for ∼30 min to adhere macrophage-precursors. Non-adherent BM cells were reseeded in 6-well plates for the differentiation into BMDCs, with media/cytokine replacement on days 3 and 5. Day 7 BMDCs were harvested by gentle scraping on ice. Differentiated BMDCs were routinely 50–60% CD11c/CD11b^+^, CD80^hi^, CD86^lo^ and MHC-II^lo^, as assessed by flow cytometry. For antigen presentation, BMDCs were seeded at 3 × 10^4^ cells/well in 96-well-flat-bottomed plates more than 6 h prior to *Salmonella* infection. For sorts, DCs were seeded at 5 × 10^6^ cells/9 cm plate prior to infection. At 20 h post-infection, cells were washed with PBS, then antigen (HEL protein (Sigma) or HEL_46–51_ peptide (Cambridge Bioscience)) and T cells were added. T-cell hybridomas were pre-washed with DMEM, 5% FCS and were added at a 5:1 T cell:DC ratio (refer to Supporting Information Fig. 3 for titration). Culture supernatants were harvested after 24 h, frozen at −80 °C and then IL-2 quantified by ELISA using mouse IL-2 Ready-SET-Go! kits (eBioscience).

### Salmonella strains, infection and flow cytometry

*Salmonella Typhimurium* 12023 (ATCC) WT and Δ*ssa*V strains that constitutively express GFP from pFVP25.1 were grown as described previously [15]. MelJuSo, RAW264.7-CIITA and HeLa cells were infected by SPI1-invasion as described previously [15]. BMDCs and monocyte-derived macrophage were infected with stationary phase *Salmonella* (pre-opsonised in 20% normal mouse serum (PAA Laboratories) or autologous human serum, respectively, for 30 min) for 60 min at a MOI of 10:1. Where specified, *Salmonella* were HK at 65°C for 45 min prior to opsonisation. For flow cytometry, cells were harvested by scraping and incubated with appropriate antibodies, in FACS buffer (PBS, 5% FCS) at 4°C. BMDCs were incubated with Mouse Fc Block (BD Biosciences) for 5 min at 4°C prior to antibody addition. After washing, cells were fixed in 1% paraformaldehyde and analysed using an FACScan Flow Cytometer and Summit software (BD Biosciences). MHC-II surface expression was calculated as mean fluorescence of infected cells (GFP positive)/mean fluorescence of uninfected cells (GFP negative) ×100.

### Western blot

Cells were harvested using Cell Dissociation Buffer (Sigma) and lysed for 30 min at 4°C in PBS, 1% Nonidet P-40 substitute, 50 mM Tris (pH 7.5), 5 mM EDTA, 150 mM NaCl, protease inhibitor mixture (Roche Diagnostics), and 5 mM Iodoacetamide. Samples were boiled in SDS-PAGE loading buffer before protein separation by SDS-PAGE and transfer to Immobilon-P PVDF membrane (Millipore). Membranes were probed with antibodies and analysed by rapid immuno-detection using ECL. Membranes were blocked with 5% non-fat milk powder/0.05% Tween-20 for subsequent detections.

### Cryo-immunoelectron microscopy

MelJuSo cells were infected with GFP-*Salmonella* in 9 cm plates. At 12 h post-infection, cells were washed and surface HLA-DR was labelled (L243 antibody) at 4°C. After 20 min, cells were washed and returned to 37°C. After 0 and 7 h endocytosis, cells were fixed in 0.2% glutaraldehyde/2% paraformaldehyde for 2 h at RT. Fixed cells were harvested, embedded in gelatin, and cryo-sectioned using a Leica FCS [41]. Ultrathin sections (50 nm) were cut at –120°C using a Cryo-immuno knife (Diatome, Switzerland) and cells were labelled with 10 nm Protein A gold particles (Cell Biology, Medical School, Utrecht University) in PBS/1% BSA. Images were collected using a Philips/FEI CM10 electron microscope.

### Statistical analysis

Statistical analysis was performed using Graphpad Prism where *p* = 0.01 – 0.05 (*), *p* = 0.001–0.01 (**) and *p* < 0.001 (***) was considered significant.

## Abbreviations

BMDC: bone marrow-derived dendritic cell
HEL: hen egg lysozyme
HK: heat-killed
Ii: invariant chain
MVB: multi-vesicular body
pMHC-II: peptide-MHC-II
SCV: *Salmonella*-containing vacuole
SPI: *Salmonella* pathogenicity island
*S. Typhimurium*: *Salmonella enterica serovar Typhimurium*
T3SS: Type III secretion system

## Supplementary material

As a service to our authors and readers, this journal provides supporting information supplied by the authors. Such materials are peer reviewed and may be re-organized for online delivery, but are not copy-edited or typeset. Technical support issues arising from supporting information (other than missing files) should be addressed to the authors.

Figure 1: Representative gating strategies for MelJuSo, HeLa-CIITA and BMDCs (A) and flow cytometry plots showing representative MHC-II down-regulation by *Salmonella* for each HeLa-HLA-DR3 transfectant used in Figure 2 (B)

Figure 2: *Salmonella* does not down-regulate MHC-II in human monocyte-derived macrophage or a murine macrophage cell line (RAW264.7-CIITA)

Figure 3: Relationship between BMDC: T hybridoma cell ratio and IL-2 response

Figure 4: Transwell experiment showing exposure to *Salmonella* is sufficient to enhance presentation of exogenous peptide to Type B T cells
