# Supplementary material for: Salmonella polarises peptide-MHC-II presentation towards an unconventional Type B CD4+ T-cell response
Source: Eur J Immunol. 2013 Jan 14;43(4):897–906. doi: 10.1002/eji.201242983 (PMC3816330; doi:10.1002/eji.201242983)
Supplement: Supplementary file 1 [file eji0043-0897-SD1.pdf]

European Journal of Immunology

Supporting Information

for

DOI 10.1002/eji.201242983

Nicola P. Jackson, Yu Hui Kang, Nicolas Lapaque, Hans Janssen, John Trowsdale and Adrian P. Kelly

Salmonella polarises peptide-MHC-II presentation towards an unconventional Type B $CD4^+$ T-cell response

© 2013 WILEY-VCH Verlag GmbH & Co. KGaA, Weinheim

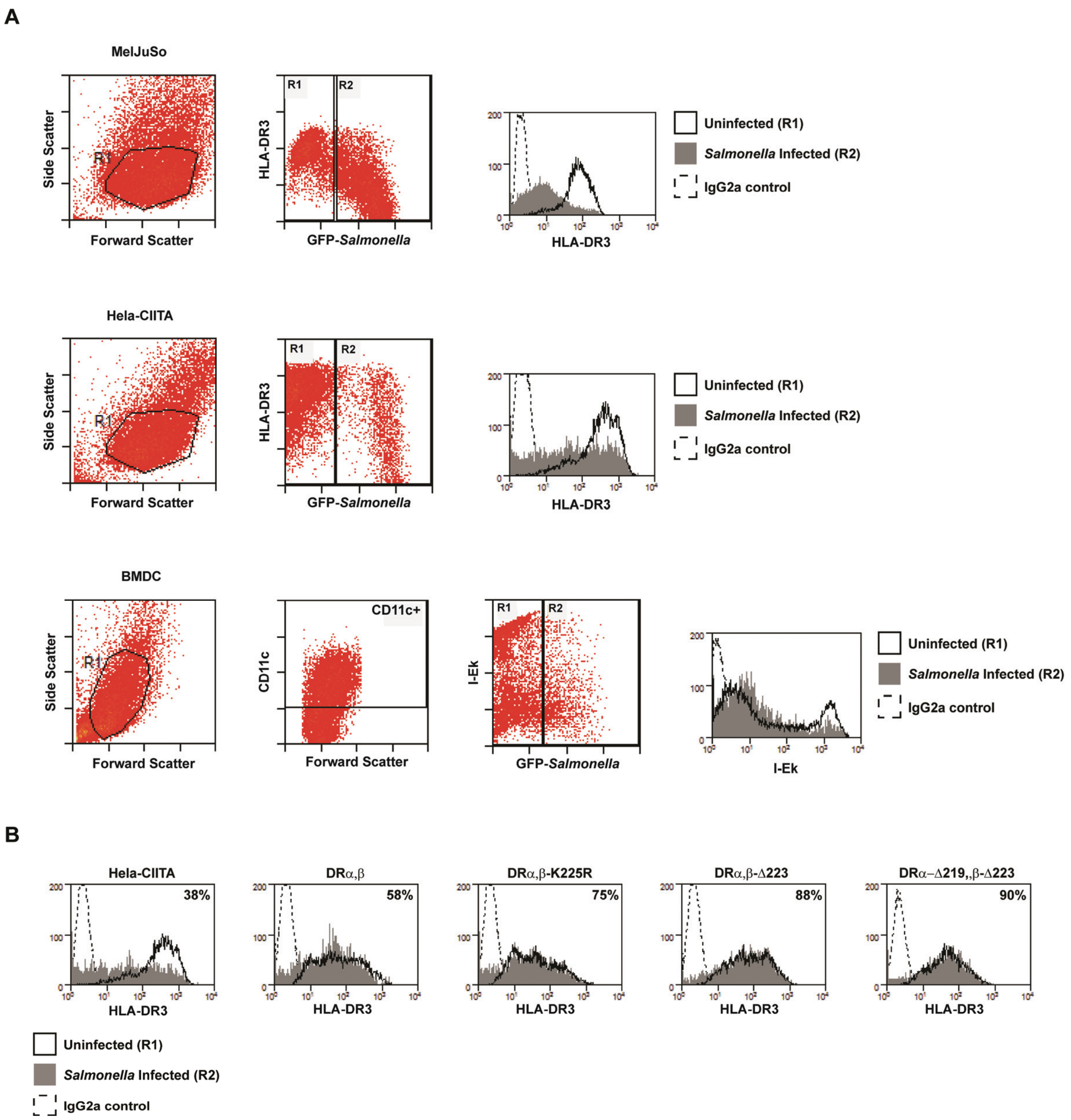

Supporting Information Figure 1: Representative gating strategies for MelJuSo, HeLa-CIITA and BMDCs (A) and flow cytometry plots showing representative MHC-II down-regulation by *Salmonella* for each HeLa-HLA-DR3 transfectant used in Figure 2 (B)

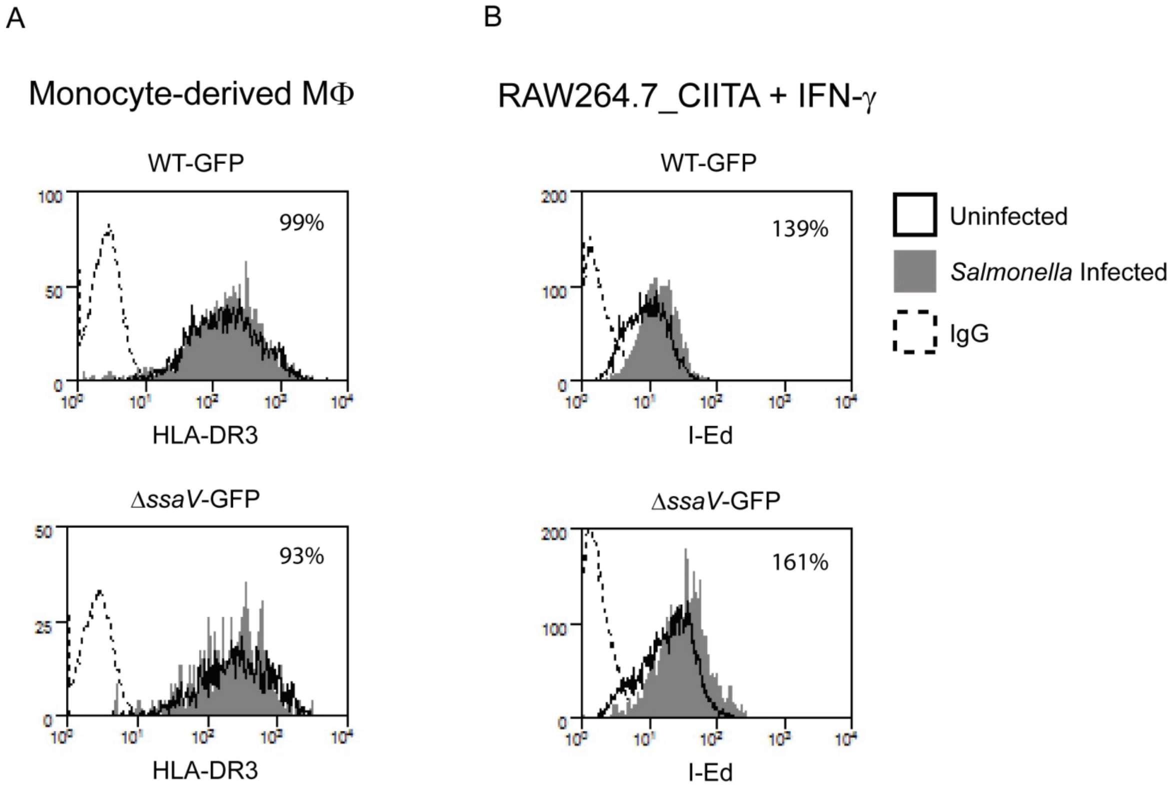

Supporting Information Figure 2: *Salmonella* does not down-regulate MHC-II in human monocyte-derived macrophage or a murine macrophage cell line (RAW264.7-CIITA)

Monocyte-derived macrophage (MΦ)(A) or IFN-γ-treated RAW264.7-CIITA cells (B) were infected with opsonised WT or SPI2-deficient (Δ*ssa*V) GFP-*S*. Typhimurium at an MOI of 50 and HLA-DR3 (L243 reactivity)(A) or I-E^d (14.4.4s reactivity)(B) surface expression was compared in infected (GFP positive) and uninfected (GFP negative) cells by flow cytometry. Histograms show HLA-DR3 (A) or I-E^d (B) surface expression in infected (shaded) or uninfected (clear) monocyte-derived MΦ (A) or RAW264.7-CIITA cells (B) from a representative of four independent experiments. Isotype controls are shown as a dashed line.

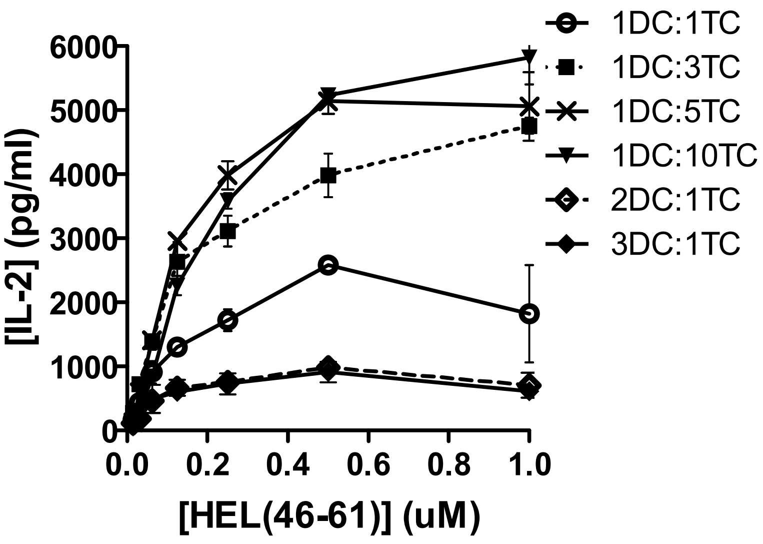

Supporting Information Figure 3: Relationship between BMDC: T hybridoma cell ratio and IL-2 response

BMDCs (in triplicate) were incubated with $HEL_{46\text{-}61}$ peptide and Type B $CD4^+$ T hybridoma cells (11A10) at different BMDC: T cell ratios to determine the optimal ratio for infection studies. After 24 h, culture supernatants were harvested and T cell activation was quantified by IL-2 ELISA. Graph shows mean IL-2 concentration from a representative of three independent experiments. Error bars represent SD.

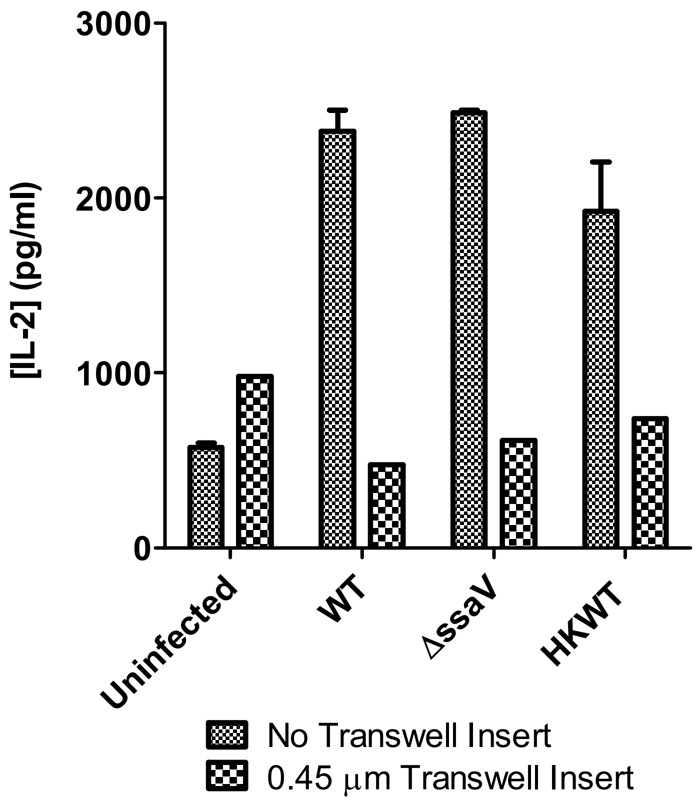

Supporting Information Figure 4: Transwell experiment showing exposure to *Salmonella* is sufficient to enhance presentation of exogenous peptide to Type B T cells

BMDCs (in triplicate) were infected with opsonised WT, SPI2-deficient (Δ*ssa*V) or HK WT GFP-*S.* Typhimurium (MOI 10). For antigen presentation, BMDCs were incubated with 0.5 μM $HEL_{46\text{-}61}$ peptide and Type B $CD4^+$ T hybridoma cells (11A10) at a ratio of 5 T cells: 1 BMDC. Where indicated, infected BMDCs were separated from fresh BMDCs and T hybridoma cells using a 0.45 μm transwell insert (Millicell-HA, Millipore). After 24 h, culture supernatants were harvested and T cell activation was quantified by IL-2 ELISA. Graph shows mean IL-2 concentration from a representative of three independent experiments. Error bars represent SD.
